# Real-Time Analysis of Gas Metabolites of Gut Microbiota in Rats Using Tunable Diode Laser Absorption Spectrometry

**DOI:** 10.3390/s26216919

**Published:** 2026-10-31

**Authors:** Ivan O. Avdeev, Yakov Ya. Ponurovskii, Denis A. Shashurin, Dmitry B. Stavrovskii, Oleg S. Medvedev

**Affiliations:** 1 Lomonosov Moscow State University, Medical Research-Educational Institute, Faculty of Basic Medicine, 119991 Moscow, Russia; avdeevio@my.msu.ru (I.O.A.); oleg.omedvedev@gmail.com (O.S.M.); 2 Chazov National Medical Research Center for Cardiology, Smirnov Institute of Experimental Cardiology, 121552 Moscow, Russia; 3 Prokhorov General Physics Institute of the Russian Academy of Sciences, 119991 Moscow, Russia; jakov@nsc.gpi.ru (Y.Y.P.); stavr@nsc.gpi.ru (D.B.S.); 4 Space Research Institute, 117997 Moscow, Russia

**Keywords:** gut microbiota, tunable diode laser spectrometry, hydrogen, methane, respiratory quotient, real-time monitoring, lactulose, gas metabolites

## Abstract

The paper presents the results of a pilot application of a system for continuous real-time monitoring of gas metabolites of the intestinal microbiota based on tunable diode laser spectrometry in combination with selective hydrogen and oxygen sensors in rats. Experiments were carried out to record the overall formation of metabolites under normal conditions and after carbohydrate load tests, as well as to monitor hydrogen and methane concentrations in exhaled air only and to assess the respiratory quotient. The results demonstrate the technical feasibility of using the system to study the patterns in gas metabolite dynamics to evaluate the functional activity of the intestinal microbiota.

## 1. Introduction

Over the last few years, increasing attention has been paid to studying the role of microorganisms in the gastrointestinal tract, or intestinal microbiota, of mammals and their ability to generate metabolites that can regulate numerous biological processes in the host organism, as well as protect it from pathogens through colonization resistance [1].

The variety and number of microorganisms in the gastrointestinal tract are the highest among all microbiomes of the human body. The number of microbial cells along the gastrointestinal tract is heterogeneous. In the stomach, it is 10^2^–10^3^ bacteria/mL, which is apparently due to the high acidity of the contents. In the duodenum and upper parts of the small intestine, this value reaches 10^3^–10^6^ bacteria/mL, which is defined by a relatively high rate of passage of contents. In the lower parts of the small intestine, it is 10^7^–10^9^ bacteria/mL. The highest concentration of microorganisms is found in the colon, where it reaches 10^10^–10^12^ bacteria/mL [2,3]. A variety of factors can alter the normal intestinal microbiota, causing intestinal dysbiosis, which is, in turn, associated with several diseases, including inflammatory bowel diseases, malignancies, metabolic, cardiovascular, and neuropsychiatric disorders [4,5,6,7].

The microorganisms of the gut microbiota ensure the breakdown of complex carbohydrates and other compounds entering the body with food. Their fermentation results in the formation of many low-molecular-weight compounds, including vitamins, minerals, hormones, short-chain fatty acids, gas metabolites such as hydrogen, methane, and hydrogen sulfide, and other products [8,9]. Many of them can be detected in blood and other body fluids, which is essential both for assessing the state of the microbiota and for diagnosing various pathologies. In particular, the formed gas metabolites enter the portal venous system and are transported to the liver and lungs, making it possible to detect them in exhaled air [10].

The determination of exhaled gases is used to diagnose many gastroenterological diseases, including small intestinal bacterial overgrowth syndrome (SIBO), impaired carbohydrate digestion, and changes in orocecal transit time. The number of studies is increasing, but the clinical interpretation of their results currently remains difficult because of the lack of uniform standards for preparation, indications, and methods of implementation [11]. To solve this problem, several medical associations, including the European Association for Gastroenterology, Endoscopy and Nutrition and the European Society of Neurogastroenterology and Motility, have proposed consensus recommendations [12,13,14] for conducting exhaled-air studies and using their results in clinical practice. Despite this, in-depth studies of exhaled-air metabolites using various methods are continuing, aimed at identifying standardized and optimal approaches [15].

One of the diagnostic tasks arising from the study of gas metabolites of the microbiota is to accurately determine the time at which their concentration begins to increase in exhaled air after the patient receives a load of carbohydrate such as lactulose. A rapid, significant increase in the hydrogen content of exhaled air within 90 min after such a load, indicating the presence of a large amount of microbiota in the small intestine, is considered a diagnostic sign of SIBO. According to the North American Consensus statement, such a test is considered positive in the case of an early increase in hydrogen of 20 parts per million (ppm) [11]. Currently existing methods of breath testing, which involve the collection of discrete samples of exhaled air and their subsequent analysis by gas chromatography, allow measurements only once every 15–20 min [16]. This limits the ability to accurately determine the onset of metabolite growth and assess the further kinetics of their formation. To solve this problem, it is necessary to use analytical methods that allow continuous analysis of exhaled air in real time.

Another area of gas metabolite research that currently remains vastly unattended is their detection directly in flatus. Their concentrations in this medium are significantly higher than in exhaled air (where they appear only after systemic distribution and several tissue barriers, i.e., intestinal wall, liver, and alveolar walls) and reflect the true rate of their formation by gut microbiota [17]. However, collection of flatus requires either invasive manipulations to obtain samples from the rectum or long-term sampling of air in the perianal area to collect flatus during episodes of flatulence. The former creates significant inconvenience and burden for patients, while the latter again requires analytical methods allowing long-term continuous monitoring in real time. A few systems based on different detection principles have been proposed to address this gap, but as of now their capabilities are limited to clinical research with a focus on the dynamics of flatus formation rather than experimental studies of specific metabolic activity of gut microbiota [18,19]. Over the past decade, optical methods based on diode laser spectroscopy (DLS) have emerged as a promising approach for breath analysis in experimental and clinical settings. Techniques such as tunable diode laser absorption spectroscopy (TDLAS), cavity ring-down spectroscopy (CRDS), cavity-enhanced absorption spectroscopy (CEAS), and photoacoustic spectroscopy demonstrate high sensitivity, allow real-time analysis, are non-invasive, and are able to detect key gases required for research and diagnostic purposes such as CH_4_, NO, NH_3_, and CO_2_ isotopes in exhaled air [20,21,22,23]. However, existing DLS-based systems have limitations that restrict their application in complex physiological studies. Firstly, they are typically designed for the detection of one or two target gases, which does not allow simultaneous monitoring of the complex multi-component profiles characteristic of gut microbial metabolism [24]. Secondly, such systems still focus predominantly on respiratory gases, whereas the total gas profile, including both respiratory and flatus-related gases, remains largely unexplored.

In the current study, we assessed the possibility of long-term monitoring of concentrations of gas metabolites of gut microbiota in exhaled air and flatus in laboratory animals using the continuous monitoring system based on DLS (TDLAS) combined with selective H_2_ and O_2_ sensors initially described in [25]. For the purposes of the study, the system was redesigned to allow measurements using unrestrained animals kept in normal conditions with access to food and water. Hydrogen sensors were also replaced to increase their sensitivity and response rate to allow measurement of H_2_ concentrations outside of flatulence episodes. The capabilities of the system were assessed in different experimental settings, including 24 h monitoring of the total gas profile in normal conditions and after carbohydrate loads, as well as long-term continuous and intermittent monitoring of exhaled air only.

## 2. Materials and Methods

### 2.1. DLS System

The study was conducted using a gas analysis system based on a custom DLS (TDLAS) spectrometer with multipass Herriott cells and three near-infrared diode lasers to measure the concentrations of CO_2_, CH_4_, and H_2_O, as well as selective O_2_ sensors and H_2_ sensors [25,26,27,28,29].

**Figure 1:**
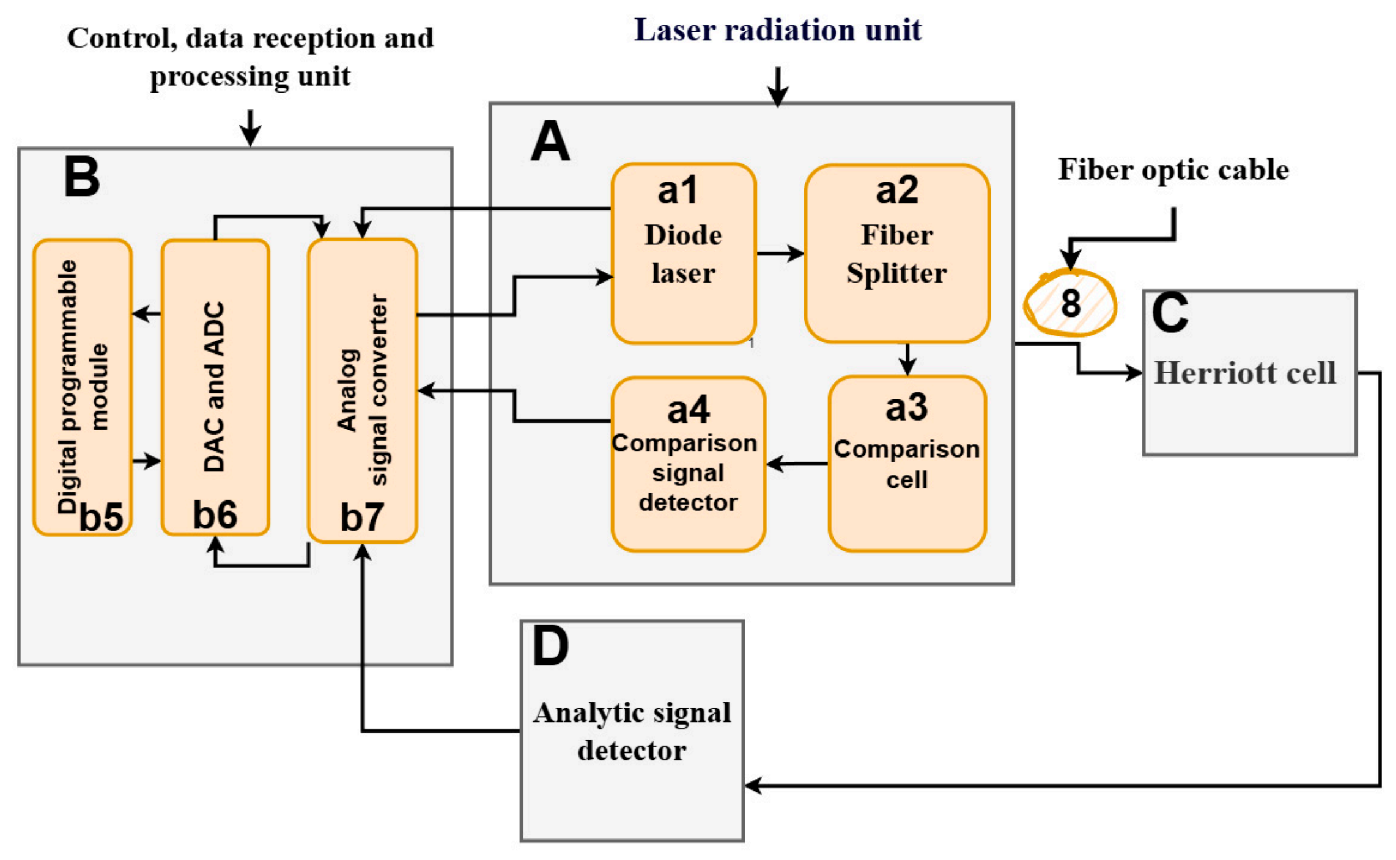
Block diagram of the DLS component of the system including: **A**, laser radiation unit; **B**, control, reception, and processing unit; **C**, multipass optical Herriott cell with fiber input; **D**, analytical signal detector. Units **A** and **B** include: a1, diode laser module; a2, fiber splitter; a3, comparison cell and Fabry–Perot interferometer; a4, comparison signal detector; b5, digital programmable module; b6, DAC/ADC module; b7, analog signal converter; 8, fiber-optic cable.

A block diagram of the DLS component of the system is shown in Figure 1. It is based on an evacuated multipass Herriott cell (C) designed by Prokhorov General Physics Institute of the Russian Academy of Sciences (Moscow, Russia), made of stainless steel, with an optical path length of 35 m and a volume of 2 L. The cell thermal stabilization system maintains a stable temperature in the cell cavity of 48 ± 1 °C to prevent moisture condensation.

Diode lasers (a1) (LD-PD Inc., Woodlands Loop, Singapore), controlled by a digital programmable module (b5) based on the NI USB-6343 I/O board (National Instruments, Austin, TX, USA) with digital-to-analog (DAC) and analog-to-digital (ADC) conversion modules (b6), generate a sequence of identical pulses at wavelengths corresponding to the absorption lines of the detected gases. The diode laser emitter is tuned to a given wavelength range using a reference signal detector (a4) (Beijing Lightsensing Technologies Ltd., Beijing, China) as the temperature of the Peltier thermoelectric element on which the diode laser is mounted changes. The electrical signal from the temperature sensor on the Peltier thermoelectric element enters the digital programmable module (b5) through the analog signal converter module (b7), where it is normalized to a unified current signal. The normalized signal is then fed to the ADC (b6), which performs sampling and quantization, converting the analog signal into a digital one. Signal processing is carried out using a personal computer with the LabVIEW 18.0 software environment (National Instruments, Austin, TX, USA). Using the DAC (b6), the digital code is converted back into an analog control signal, thereby closing the automatic temperature control circuit of the Peltier thermocouple.

The main part of the diode laser radiation from the fiber splitter (a2) enters the multipass optical cell (C) through the fiber-optic cable (8), where the analyzed gas mixture is supplied. After passing through the cell, the diode laser radiation is focused on the photodetector of the analytical signal detector (D) (Beijing Lightsensing Technologies Ltd., Beijing, China). The signal from the detector is then amplified, digitized using the ADC (b6) and the analog signal converter module (b7), and transmitted to a personal computer to calculate the concentration of the detected gas. Electric synchronization of the laser modules is software-supported using multifunctional NI USB-6343 boards. Time distribution of signals from lasers in the receivers of the analytical channel and the reference channel is performed using time multiplexing of diode laser pumping current pulses. As confirmed during initial validation of the system, after accumulation and averaging of the sample of 64 realizations of the measured signal, the signal-to-noise ratio in the multipass cuvette is better than 5 × 10^5^.

**Figure 2:**
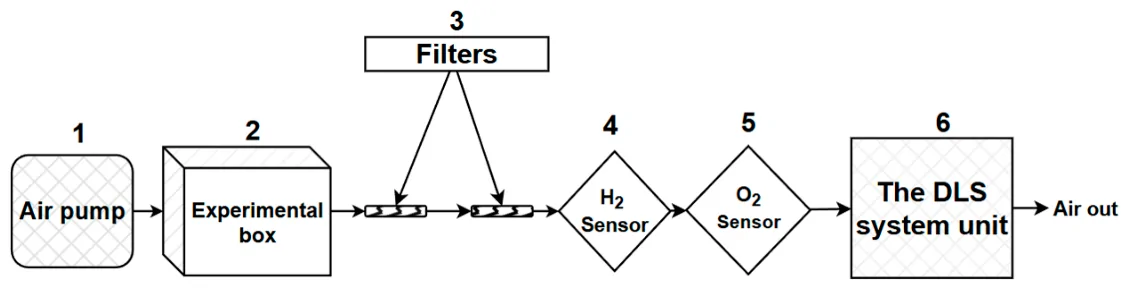
Diagram of the unit for measuring exhaled H_2_, CH_4_, CO_2_, and O_2_ biomarkers in small animals.

The complete setup and the flow scheme of the analyzed gases are shown in Figure 2. The setup consists of an air pump (1), which supplies ambient air to the experimental box (2) containing the animal. The air flow rate is constantly monitored and controlled using a rotameter. The analyzed gas mixture then passes from the experimental box through moisture-absorbing and carbon filters (3) and sequentially enters the metal–dielectric–semiconductor (MDS) hydrogen sensor (4) (Moscow Engineering Physics Institute, Moscow, Russia), with a measuring range of 0.1–1000 ppm. The sensitive element and basis of the sensor is an MDS structure, which is a capacitor consisting of a silicon wafer, a silicon dioxide dielectric layer, and a palladium metal electrode (Pd–SiO_2_–Si). When molecules of the studied gas are deposited on the surface of the palladium electrode, a change in electrical capacitance is observed and converted into an electrical signal. The measured gases then enter the electrochemical oxygen sensor (5) (Oksonium Ltd., St. Petersburg, Russia), whose operating principle is based on a change in electrical voltage proportional to the partial pressure of oxygen in the air of the working area. The signals from the electrochemical sensors are digitized and transmitted to a personal computer for calculating gas concentrations, data processing, and visualization. The flow is then directed to the DLS component described above (6).

The detection limits and working concentration ranges of the DLS system are summarized in Table 1.

The other key technical parameters of the system that determine its temporal resolution and measurement stability include the signal acquisition rate, response time, gas transit delay through the tubing and filters, and the signal averaging method. Spectral data acquisition by the laser spectrometer is performed at a rate of at least several hundred hertz, providing a high temporal resolution of the raw signal at the level of 10 milliseconds. The final temporal resolution for concentration profiles is determined by the gas exchange rate in the multipass cell and the signal averaging time. The response time of the laser part of the system, determined by the gas exchange rate in the multipass cell with a volume of 2 L at a flow rate of 1.7 L/min, is under 20 s. For the MDS hydrogen sensor, the response time is in the range of 30–40 s, and for the electrochemical oxygen sensor, it is 10 s, which is consistent with the specifications of such sensors. The gas transit delay from the animal chamber to the detectors through the tubing and filters was measured experimentally and was approximately 30 s.

To reduce high-frequency noise and ensure measurement stability during long-term experiments, all concentration values are obtained using an accumulation and averaging procedure. The system uses built-in accumulation and averaging of the signal from 16 consecutive gas concentration measurements, which takes 0.85 s. Depending on the analysis task, the time grid step (the recording interval of averaged values) can vary from several tens of seconds to 1 min or more, allowing a flexible balance between temporal resolution and signal stability. No additional smoothing or digital filtering is applied.

Calibration of the DLS system for the current study was performed using certified gas mixtures based on inert and permanent gases, containing hydrogen and methane at concentrations of 5 ppm and 50 ppm (Monitoring LLC, St. Petersburg, Russia). The calibration was carried out before each experiment by introducing 5 L of calibration gas into the DLS system. In-study validation of the system was performed by comparing measured H_2_ and CH_4_ concentrations with results of analysis of the air samples obtained from the output tube of the animal chamber using a gas chromatograph (GC) with solid-state detectors, QuinTron BreathTracker SC Analyzer (QuinTron Instrument Company, Inc., Milwaukee, WI, USA). The samples for these reference analyses were collected approximately hourly during working hours.

Before the start of the animal experiments, the system was tested for 24 h under conditions fully identical to the experiment to assess its stability. The results showed that the daily signal drift, determined from the concentration trends, did not exceed the threshold values and amounted to 2% of the background level for hydrogen and methane. Reproducibility was confirmed by repeating the same protocol on different days.

### 2.2. Animal Studies

Pilot experiments on the registration of gas metabolites of the microbiota were carried out on male Wistar rats with conventional microbiological status weighing 200–250 g, obtained from the branch of the State Scientific Center of the Russian Federation—Institute of Biomedical Problems of the Russian Academy of Sciences, Khimki, Russian Federation. Outside the experiment, the animals were housed in a vivarium with a standard 12 h light cycle and unlimited access to food and water.

Studies with carbohydrate load tests included intragastric administration of lactulose to boost and standardize the production of gaseous metabolites by gut microbiota. Lactulose (LLC “Slavyansky Food Processing Plant,” Moscow, Russia) was administered at a dose of 0.2 g/100 g body weight dissolved in 2 mL of water.

#### 2.2.1. Measurement of the Total Release of Gas Metabolites

Baseline performance assessment of the system in the study conditions was conducted by 24 h monitoring of gaseous metabolites, including metabolites released both through respiration and as flatus, in individual rats (*n* = 2) receiving standard chow. The animal was placed in a sealed plastic experimental box with a volume of 1.3 L connected to the measuring system. A water dispenser and feeder were provided in the box to allow long-term housing of the animal for continuous monitoring up to 24 h. The box was ventilated using a membrane pump at a rate of 1.7 L/min to ensure a stable CO_2_ concentration in the range of 0.5–0.6%. The air supplied was filtered by an H13 HEPA filter and temperature-controlled at 25 °C. The measurements continued for 24 h.

During the study, two types of measurements of total release of gaseous metabolites after carbohydrate load were performed. In the first experimental series (n = 3), the animal remained in the box for 4 h. Four-hour measurements were performed twice: first under standard feeding conditions, and subsequently after fasting for 12 h followed by lactulose administration. In the second series (*n* = 2), the animal remained in the box for 24 h after intragastric administration of lactulose. For the first 6 h, the animals had no access to food; subsequently, they were given unlimited access to standard feed for continuation of the daily measurement.

#### 2.2.2. Measurement of Gas Metabolites Released Through Respiration

**Figure 3:**
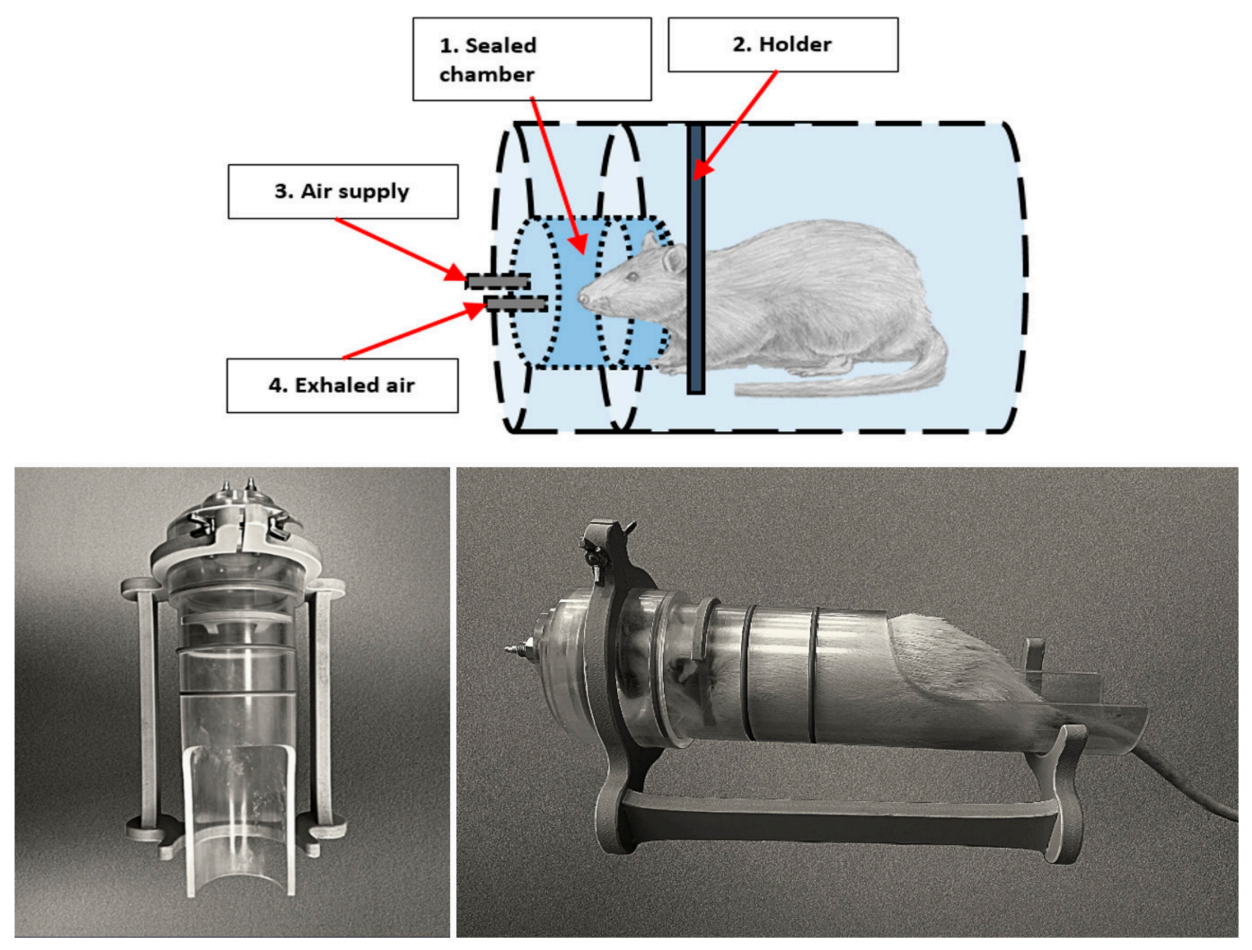
Schematic diagram and photo of the restrainer.

To register metabolites released exclusively through respiration rather than flatus, a custom restrainer isolating the head of the animal was used (Figure 3). Animals (*n* = 3) were first adapted to the restrainer for one week. Each animal was fixed in the restrainer with a holder of suitable size in the neck area without causing discomfort to the animal. The anterior part contained an almost completely sealed chamber, in which air was circulated by a membrane pump at a rate of 1.4 L/min to ensure a stable carbon dioxide concentration in the range of 0.5–0.6%. The air from the chamber was supplied to the measuring system.

Before the study, the animals were kept fasting for 12 h. To limit the duration of animal restraint, the measurements were performed intermittently instead of continuously, alternating between animals. The study began with the determination of baseline concentrations of gas metabolites in exhaled air during a period of 10 min. Then, lactulose was administered. Further measurements of metabolite concentrations were performed at approximately 2 h 20 min, 4 h 15 min, 5 h 10 min, and 6 h 10 min. All three animals were tested sequentially within the same measurement session. Each measurement lasted 10 min per animal, with approximately 20 min intervals between individual animals. Between the measurements, the animals were kept in their standard housing cages with unlimited access to water but without food.

### 2.3. Statistical Analysis

Data are presented as the area under the curve (AUC) for hydrogen and methane concentrations. The AUC was calculated using the trapezoidal method in the LabVIEW environment (National Instruments) with the Integral x(t).vi function. The AUC results are presented in ppm·h. In the restrainer experiments, the baseline level was determined individually for each peak based on the recording segment immediately preceding it. Integration was performed over fixed time intervals (8 min) corresponding to the middle of each measurement. In the sealed chamber experiments (4-h measurements), the baseline level was determined from the background recording obtained in the chamber without an animal and was subtracted from the time series. Integration was performed over the entire duration of the experiment.

Due to the pilot nature of the study and the small sample size (*n* = 2–3 per group), only descriptive statistics were applied and individual responses were plotted.

## 3. Results and Discussion

### 3.1. Measurement of the Total Release of Gaseous Metabolites

#### 3.1.1. Twenty-Four-Hour Monitoring

**Figure 4:**
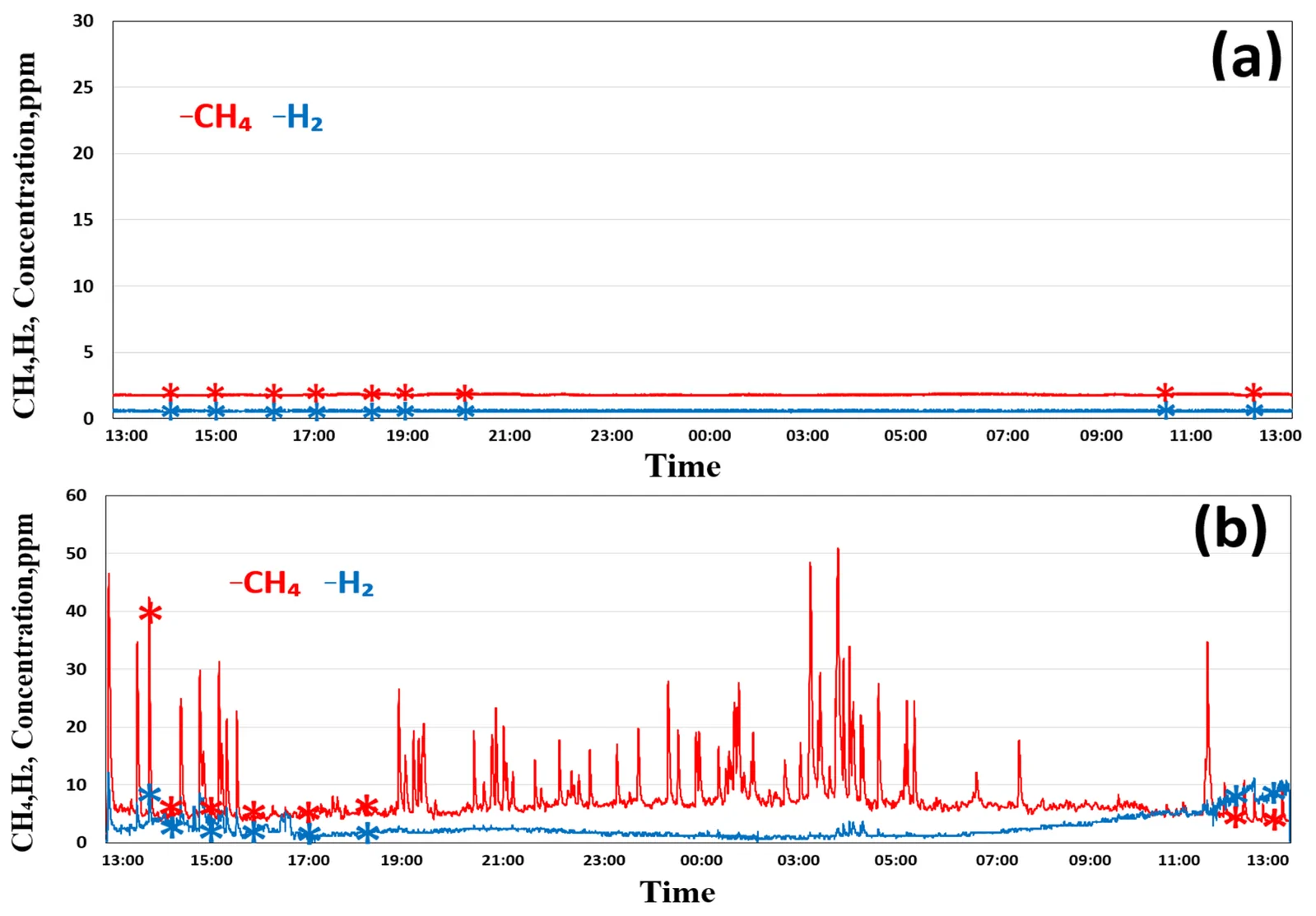
Twenty-four-hour continuous recording of CH_4_ and H_2_ concentrations in a sealed chamber: (**a**) background recording without an animal; (**b**) recording with a rat on a standard diet. Asterisks indicate results of reference measurements.

Figure 4 presents the results of 24-h continuous recording of methane and hydrogen concentrations in a sealed chamber.

The upper panel (a) corresponds to the background recording performed without an animal. This measurement was carried out to assess the system background and to exclude artifacts that could be mistakenly interpreted as a biological signal. Throughout the entire 24-h period, the methane concentration remained stable at 1.7–1.9 ppm, and the hydrogen concentration at 0.4–0.6 ppm that was in line with their normal concentrations in room air. No peaks or increases in gas metabolite concentrations are present on the curves, which confirms the absence of instrumental effects.

The lower panel (b) shows the recording obtained with an animal on a standard diet. Here, in contrast to the background recording, numerous methane peaks reaching 30–50 ppm were observed. Considering the absence of instrumental artifacts as confirmed above, these peaks could be attributed to the release of intestinal gases, i.e., flatulence. The peaks have a characteristic shape: a rapid rise and a relatively fast decay, which is typical of short-term episodes of gas release. The amplitude of the peaks varied from small emissions up to 10 ppm to the highest concentrations of 20–50 ppm, potentially reflecting the natural irregularity of fermentation and intestinal motility processes. The measured hydrogen concentration remained considerably lower (at 1–2 ppm), potentially indicative of functional prevalence of methanogenic microbiota and fast conversion of H_2_ into CH_4_. The hydrogen concentration records also contained fewer flatus peaks, probably due to lower H_2_ concentration in intestinal gas or limited temporal resolution of the MDS hydrogen detector compared to DLS.

The results of the reference measurements (marked in the figure by asterisks) by gas chromatography were aligned with the data obtained from the DLS system, accounting for the specifics of both systems. During background recording without an animal, all GC measurements (*n* = 9) returned zero values for H_2_ concentrations and 1 ppm for CH_4_ concentrations due to the QuinTron BreathTracker’s lower detection threshold (declared as 2 ppm) for both gases. The results of DLS and reference measurements during the animal experiment (*n* = 8) are presented in Table 2. In all cases, the differences were under 1 ppm, corresponding to the QuinTron BreathTracker resolution.

Over the 24-h period, notable changes in methane and hydrogen concentrations were observed. In the initial period from 13:00 to 15:00, an increase in both gases was noted, with active flatus peaks of methane above 40 ppm and hydrogen above 10 ppm. This was likely associated with food intake before the start of the recording and the ongoing fermentation in the intestine. Then, from 15:00 to 19:00, the flatulence intensity decreased, the methane concentration lowered to 4–5 ppm, and the hydrogen concentration decreased to 1 ppm, with single flatus peaks up to 3 ppm. In the interval from 19:00 to 21:00, an increase in hydrogen to 2 ppm was observed; the methane concentration remained at the previous level, but the onset of active flatus activity was noted. This was likely related to the start of food intake during the evening and its subsequent fermentation, with initial formation of H_2_. The period after 21:00 was characterized by a gradual increase in methane concentration and flatus peaks starting at 6–7 ppm and 15–25 ppm respectively before midnight and reaching 8–9 ppm and up to 50 ppm respectively during the night. The hydrogen concentration remained stably low, except for small flatus peaks up to 3 ppm. The decrease in hydrogen with a simultaneous increase in flatulence and methane concentration indicates the active conversion of hydrogen to methane by methanogenic archaea. In the period from 07:00 to 13:00, a gradual increase in hydrogen to 9 ppm was observed, while methane decreased to 4 ppm. The frequency of peaks was reduced, and they appeared only between 12:00 and 13:00. The reasons for this hydrogen increase require further assessment because they do not fully correlate with the anticipated daily dynamics, and the results of the first hours of measurement reflect a similar period of the day.

**Figure 5:**
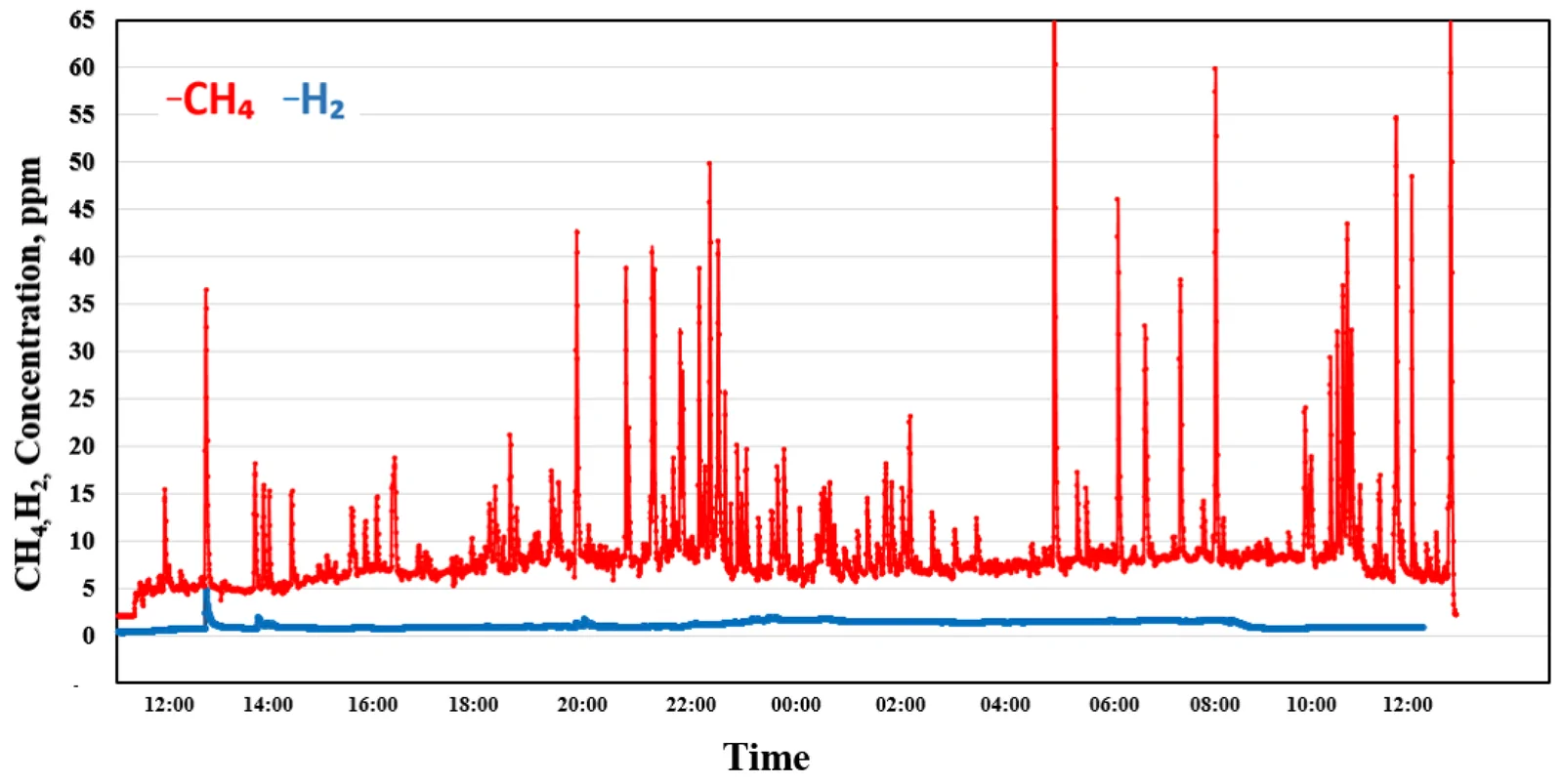
Results of daily registration of concentrations of gas metabolites of the microbiota after the administration of lactulose.

An example of the results of daily measurements of methane and hydrogen concentrations in exhaled air and flatus in an animal after lactulose administration after fasting is shown in Figure 5. At the beginning of the study, the concentrations of methane and hydrogen were approximately 5 ppm and <0.5 ppm, respectively. After the carbohydrate load, a gradual increase in methane concentration to approximately 9 ppm and a slight increase in hydrogen concentration were noted, with a gradual increase in the frequency of flatus peaks. This potentially reflects intestinal fermentation of lactulose with functional predominance of hydrogen-to-methane conversion by methanogenic archaea of the microbiota. Notably, in the recording obtained with an animal on a standard diet without a carbohydrate load (Figure 4b), the time interval from approximately 16:00 to 19:00 was characterized by the absence of methane concentration increases and flatus peaks. This can be attributed to the absence of lactulose administration, which enhances intestinal motility and fermentation, in the latter case.

After the animal gained access to feed at 18:00, a further increase in flatus formation and flatulence was observed. This could be related to an increase in the intensity of peristalsis as a result of food intake after a 24-h fasting period, as well as the rapid entry of a significant amount of partially digested contents into the large intestine, which, in turn, increases the amount of available nutrients for intestinal microorganisms [30]. Signs of completion of the lactulose digestion process, namely a decrease in methane concentration and the intensity of flatulence, were recorded approximately 8 h after the carbohydrate load, at 20:00. After the beginning of the dark phase of the light cycle at 21:00, a repeated increase in methane concentration in exhaled air and an increase in flatulence were noted, which could be due to the daily cycle of the animal’s activity and nutrition, as well as the onset of fermentation of the food received 4 h earlier. Interestingly, the frequency and amplitude of flatus activity on the standard diet (Figure 4b) were noticeably lower at this time and reached a greater increase only from approximately 03:00 to 05:00. After the eleventh hour, at 23:00, and throughout the night, stabilization of the methane concentration was observed at approximately 7 ppm, with a slight but constant increase in hydrogen concentration. Such dynamics could be explained by temporary saturation of the metabolism of methanogenic archaea, leading to a decrease in the rate of hydrogen uptake by them below the rate of its formation by hydrogen-producing anaerobes. At the beginning of the next day, a decrease in methane concentration to the baseline level was noted, aligned with the anticipated completion of the digestion of the food consumed the day before.

During this experiment, the respiratory, or metabolic, quotient (RQ) was also assessed. This quotient reflects the ratio of the amount of released CO_2_ to the amount of consumed O_2_ and reflects the predominant type of energy metabolism in the body. RQ values close to 1.0 indicate the predominance of glycolysis, while a decrease in the respiratory quotient below 0.8 indicates an increase in the intensity of lipolysis and fatty acid oxidation [31].

In the current study, RQ was calculated using the formula:

(1) $$\mathrm{RQ}=\;\frac{\left[{\mathrm{CO}}_{2}\right]}{21\%-[O_{2}]}$$

where [*CO*_2_] is the measured CO_2_ concentration in percent and [*O*_2_] is the measured O_2_ concentration in percent.

**Figure 6:**
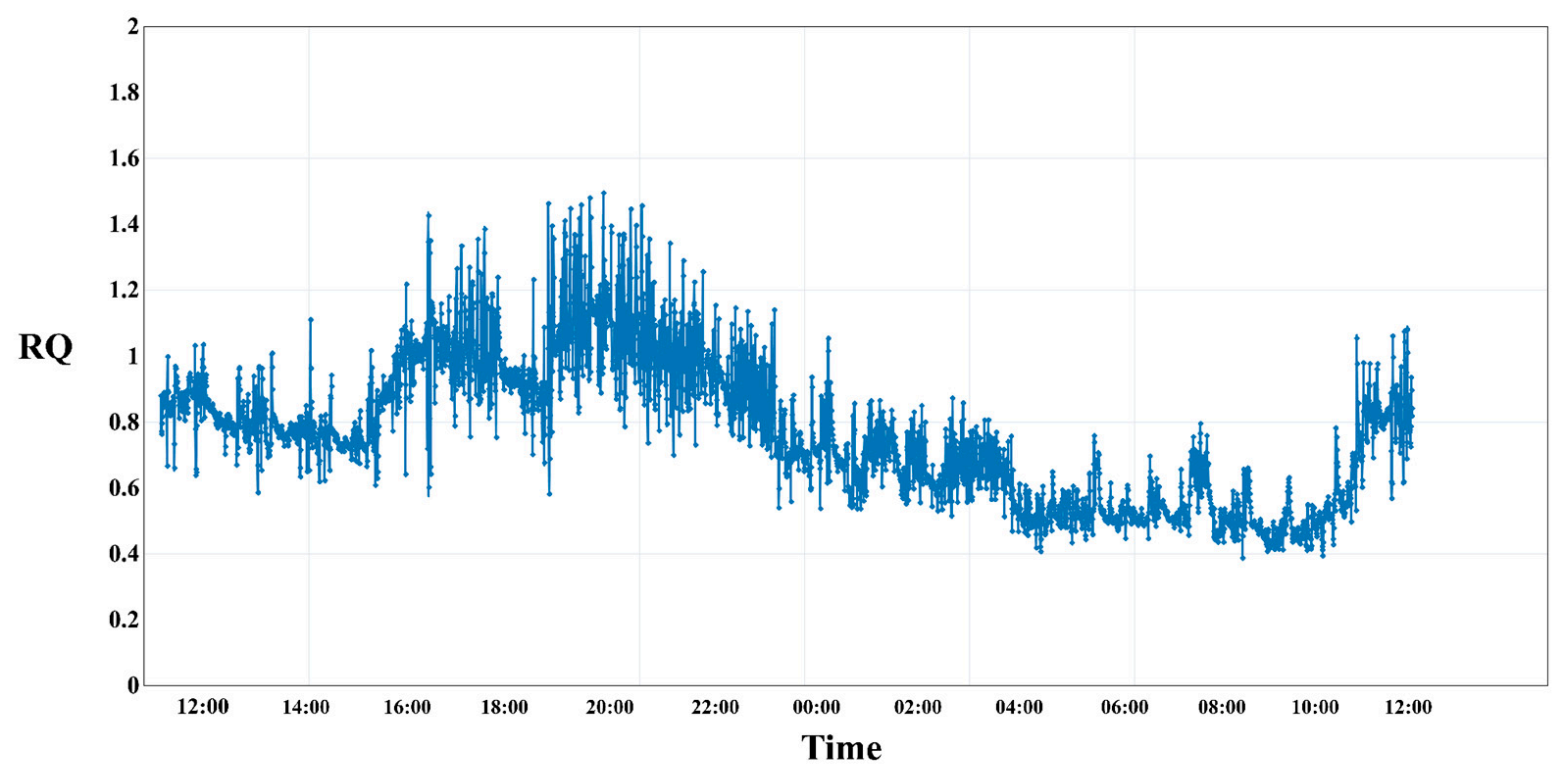
Results of daily registration of the respiratory quotient after the administration of lactulose.

At the beginning of the experiment, the animal’s RQ was approximately 0.8 (Figure 6). Three hours after the carbohydrate load, an increase in the respiratory coefficient to approximately 1.0 was noted, coinciding with an increase in methane content in exhaled air and an increase in the intensity of flatulence. The reason for the increase in RQ in this case could be limited absorption of carbohydrates after lactulose cleavage by microorganisms of the colon. After the animal regained access to food at 18:00, there was a further increase in RQ to approximately 1.1, continuing till midnight and potentially reflecting activation of glycolysis after food intake. After the end of the animal’s overnight activity period, the RQ value decreased to approximately 0.5 and returned to baseline only at the end of the daily monitoring period. It should be noted that the RQ values recorded during daily monitoring exceeded the expected range of 0.7–1.0 in some periods, with a certain correlation between the time of recording the RQ limit values and the anticipated timing of the changes in animal activity levels. This may be due to the maintenance of a constant ventilation rate in the system and the calculation of RQ based on raw measured O_2_ and CO_2_ concentrations, which does not account for the variations in their concentrations in in-flown air and the possible changes in the respiratory rate of the animal. Considering this, as well as the lack of independent verification of RQ values by standard methods such as indirect calorimetry in the context of the current study, only relative qualitative assessments of RQ rather than its quantitative evaluation can be considered at this stage. This component of the system operation algorithm requires further analysis, optimization, and validation.

#### 3.1.2. Four-Hour Monitoring After Carbohydrate Load

**Figure 7:**
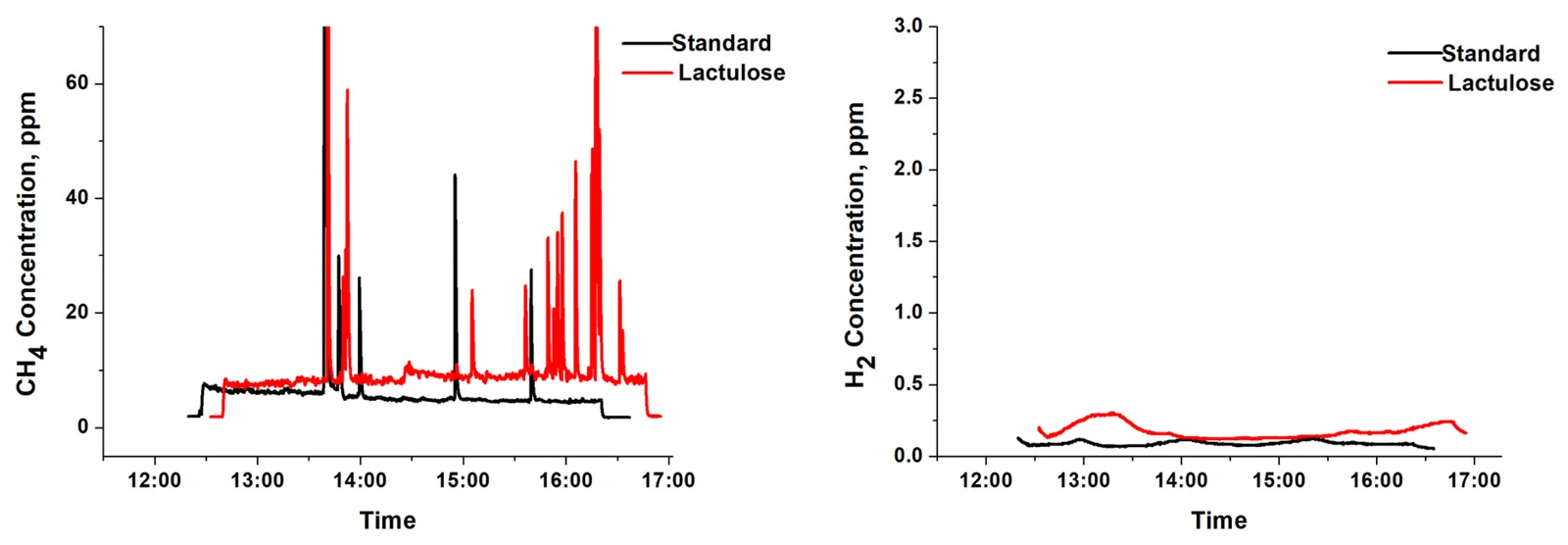
An example of the results of measuring the content of hydrogen and methane in the exhaled air and flatus of a rat placed in a sealed box for 4 h with standard feed and after the administration of lactulose.

An example of the results of continuous measurement of total gas metabolite formation within 4 h after lactulose administration is shown in Figure 7. After 1.5 h, an increase in methane concentration of >4 ppm was recorded, reaching a plateau at approximately 10 ppm. In addition, an increase in the frequency of methane peaks was observed, indicating accelerated flatus formation and increased flatulence. The increase in methane concentration and the increased intensity of flatus excretion can be explained by bacterial fermentation of lactulose in the gut. The hydrogen concentration, unlike the methane concentration, remained virtually unchanged during the first 1.5 h of measurement and was approximately 0.3 ppm. However, after the methane concentration increased, the hydrogen concentration decreased slightly, which may indicate its conversion into methane by methanogenic archaea prevailing over its initial synthesis by hydrogen-producing microorganisms [32]. No flatulence peaks were observed during hydrogen registration. This observation requires further investigation to determine whether it was truly caused by low hydrogen concentration in flatus due to its fast conversion into methane, or whether it was related to technical limitations of the system, i.e., lower response rate of the MDS hydrogen sensor compared with the DLS unit used to determine methane concentration.

**Figure 8:**
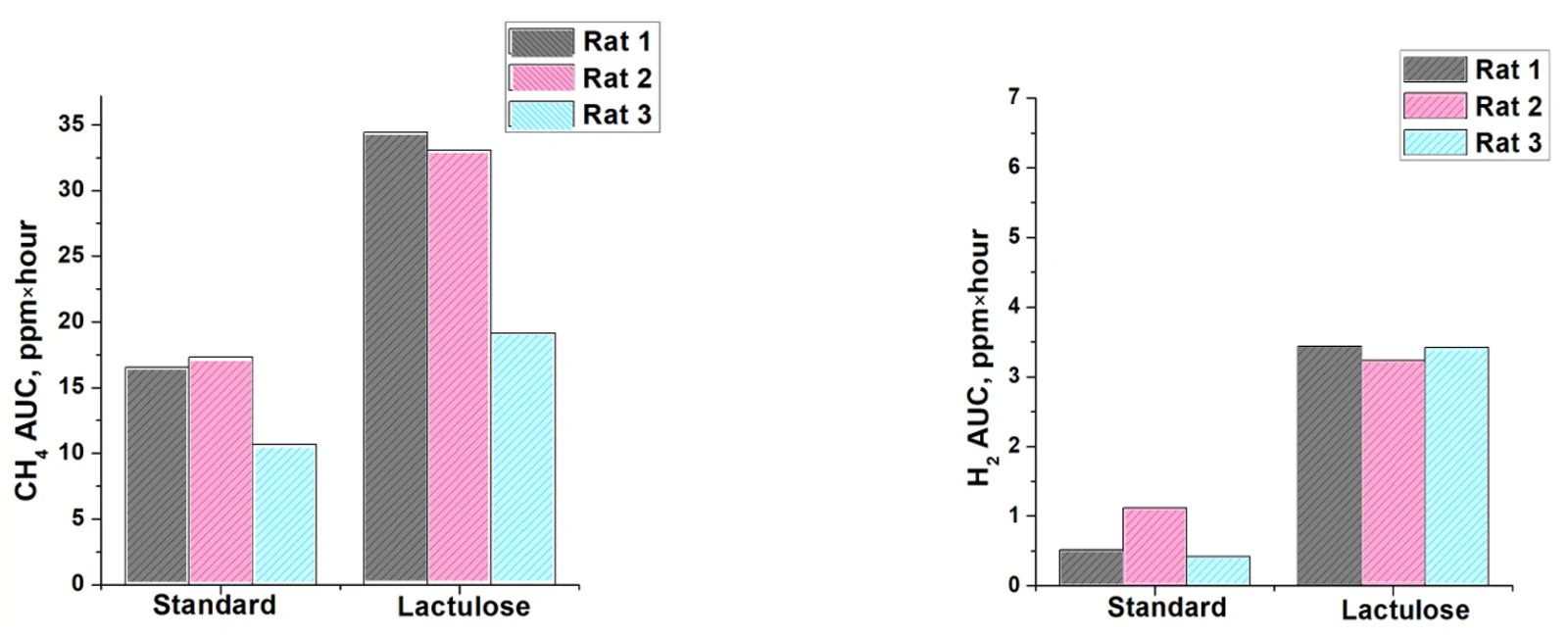
Comparison of the AUC for hydrogen and methane in three rats with continuous registration for 4 h with standard feed and after the administration of lactulose.

The results of the hydrogen and methane AUC calculations in animals under a standard diet and after lactulose administration following fasting are presented in Figure 8. Lactulose administration led to an apparent increase in methane concentrations compared with the standard diet. Individual differences in animal responses were observed, which may be due to differences in metabolism and activity levels. In particular, in Rats 1 and 2 receiving standard feed, the intensity of methane release was almost identical. However, after lactulose administration, Animal 1 showed a more pronounced increase in methane concentrations than Animal 2. Animal 3 exhibited lower methane concentrations both during standard feeding and after lactulose administration. The hydrogen formation by Animal 2 on a standard diet was markedly higher than by Animals 1 and 3. After lactulose administration, an apparent increase in hydrogen production was also noted in all three animals.

### 3.2. Measurement of Gaseous Metabolites Released Through Respiration

**Figure 9:**
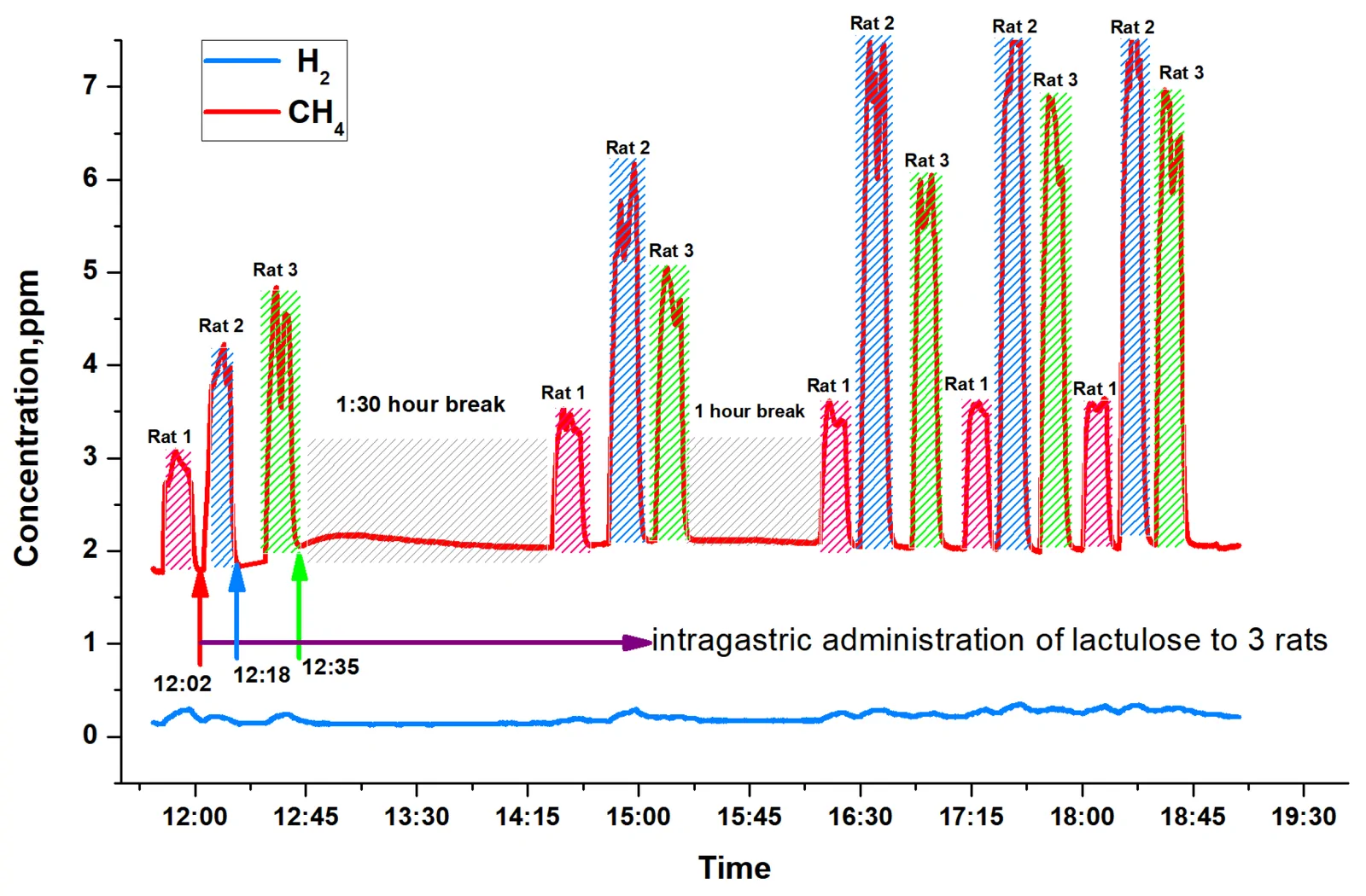
An example of the results of alternating recording of methane and hydrogen concentrations in exhaled air in three rats for 6 h. The data used to calculate the AUCs are shown by hatching (red–rat 1, blue–rat 2, green–rat 3).

**Figure 10:**
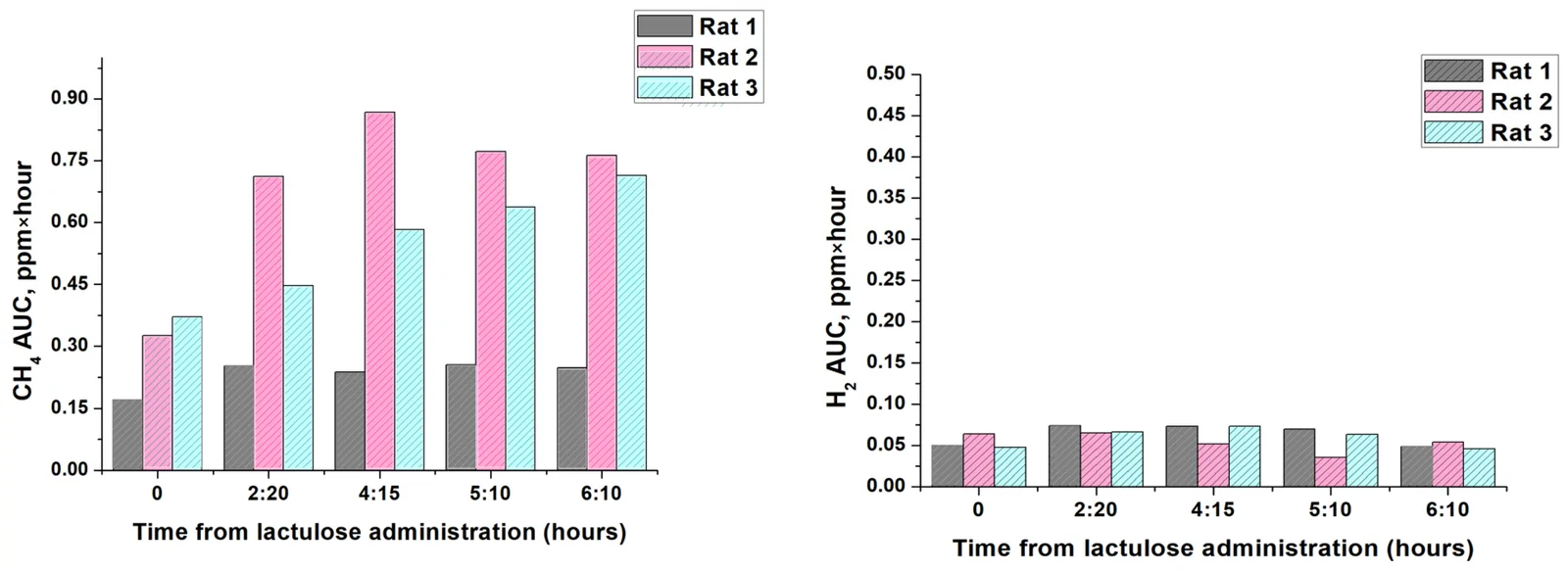
The AUCs of methane and hydrogen in exhaled air with alternating recording of 3 animals for 6 h.

Measurements of gas metabolite content in exhaled air, in contrast to measurements using a sealed container, make it possible to record the concentrations of excreted gas biomarkers in exhaled air separately from flatus. In animals treated with lactulose, a noticeable increase in methane concentration and a slight increase in hydrogen concentration in exhaled air were recorded during the six-hour observation period compared with their initial values (Figure 9 and Figure 10). Animal 1 showed a slight increase in methane concentration only approximately 2.20 h after lactulose administration relative to baseline. This interval is sufficient for the onset of lactulose fermentation in the colon. During the following 1.5 h, there was a slight decrease in methane concentration, followed by stabilization until the end of the experiment. No substantial changes in hydrogen content were observed, indicating low metabolic activity of the microbiota. Animal 2 showed an apparent increase in methane concentrations already during the second hour, followed by a decrease. The hydrogen concentration remained virtually unchanged. Considering the early CH_4_ concentration increase, this response may indicate an increased concentration of microbiota in the small intestine and, as a result, an accelerated onset of bacterial fermentation of lactulose. Animal 3 showed a gradual increase in methane concentration, reaching a peak value by the sixth hour of observation. The hydrogen concentration showed a slight increase at 2 and 4 h after the start of measurements compared with the initial level, followed by a gradual decrease. Such dynamics suggest that active bacterial fermentation of lactulose occurred only after it reached the large intestine.

## 4. Conclusions

The use of multicomponent diode laser spectrometry enables non-invasive continuous measurement of gas biomarkers of the intestinal microbiota under experimental conditions. Continuous monitoring makes it possible to record the earliest stages of methane and hydrogen formation. Analysis of long-term measurement results makes it possible to evaluate circadian changes in gas profiles reflecting circadian changes in intestinal enzymatic activity. Simultaneous analysis of gas biomarkers released through both respiration and flatus opens prospects for assessing the overall dynamics of metabolism, microbiota enzyme activity, and the state of the gastrointestinal tract. This technology may expand the possibilities for diagnosing a number of diseases caused by impaired carbohydrate metabolism and impaired intestinal wall permeability, and opens up opportunities for complex experimental studies of the functional state of the intestinal microbiota and the possibilities of its pharmacological and nutritional correction in real time.

The DLS-based system evaluated in the current study demonstrated reasonable performance in standard types of preclinical experiments requiring monitoring of CH_4_ and H_2_ in breath and flatus, including monitoring of gas profile in normal conditions, recording gas dynamics in response to carbohydrate loads, isolated monitoring of gas metabolites in exhaled air, and intermittent/alternating monitoring of gas metabolites in multiple animals. The results of the measurements were aligned with the expectations for corresponding biological processes. The calculated RQ values demonstrated dynamics generally aligned with the expectations but were outside of the anticipated ranges at certain time points, indicating the need for further evaluation of this component of system functionality. Further assessments of the system—for example, in experiments involving animals with disease models—should be done within studies aimed at addressing corresponding scientific goals rather than dedicated feasibility and performance assessment protocols.

## Figures and Tables

**Table 1:** Detection limits and working concentration ranges of the sensors used in the measuring system. All concentration values are given in volume fraction (*v*/*v*) unless otherwise stated. For gases with ranges given in %, 1% = 10,000 ppm.

Measured Gas	Detection Limit	Working Concentration Range
O_2_	0.1%	10–50% (*v*/*v*)
^12^CO_2_	20 ppm	0.005–20% (*v*/*v*)
^13^CO_2_	20 ppm	0.005–20% (*v*/*v*)
CH_4_	0.2 ppm	0.0001–1% (*v*/*v*)
NH_3_	0.01 ppm	0–50 ppm
H_2_S	0.7 ppm	1.5–20 ppm
H_2_O	0.01%	0.02–100% (*v*/*v*)
H_2_	0.1 ppm	0.2–1000 ppm

**Table 2:** Comparison of CH_4_ and H_2_ concentrations values measured by DLS and gas chromatography during animal experiment.

Measured Gas	DLS (Min–Max)	GC (Min–Max)	DLS/GC abs. Difference (Mean ± St. Dev.)
CH_4_	4.5–40.0 ppm	4.0–39.0 ppm	0.64 ± 0.34
H_2_	1.5–7.5 ppm	1–7 ppm	0.80 ± 0.23

## Data Availability

The original contributions presented in this study are included in the article. Further inquiries can be directed to the corresponding author.

## Abbreviations

The following abbreviations are used in this manuscript:

ADC	Analog-to-digital module
AUC	Area under curve
DAC	Digital-to-analog module
DLS	Diode laser spectroscopy
GC	Gas chromatography
MDS	Metal–dielectric–semiconductor
RQ	Respiratory quotient
SIBO	Small intestinal bacterial overgrowth syndrome
TDLAS	Tunable diode laser absorption spectroscopy
